# Extracellular Vesicle Derived From Mesenchymal Stem Cells Have Bidirectional Effects on the Development of Lung Cancer

**DOI:** 10.3389/fonc.2022.914832

**Published:** 2022-07-04

**Authors:** Jiayu Wang, Yiming Ma, Yingjiao Long, Yan Chen

**Affiliations:** ^1^ Department of Pulmonary and Critical Care Medicine, The Second Xiangya Hospital, Central South University, Changsha, China; ^2^ Research Unit of Respiratory Disease, Central South University, Changsha, China; ^3^ Diagnosis and Treatment Center of Respiratory Disease, The Second Xiangya Hospital, Central South University, Changsha, China

**Keywords:** mesenchymal stem cells, extracellular vesicle, lung cancer, promote, inhibition

## Abstract

Mesenchymal stem cell is a kind of pluripotent cells with the ability of self-renewal and multi-directional differentiation, which exist in bone marrow, umbilical cord blood, umbilical cord tissue, placenta tissue, adipose tissue and so on. Extracellular vesicles are membranous lipid vesicles secreted by a variety of cells and widely present in body fluids, which contain proteins, mRNA, microRNA and other substances, and are an important medium of intercellular communication. At present, more and more evidence shows that mesenchymal stem cell-derived extracellular vesicles play an important role in the development of lung cancer. Regulating the levels of proteins, RNAs and other substances in MSC-EVs and then transplanting them into patients may be a new way to alleviate the development of lung cancer. We mainly introduce the role of extracellular vesicles derived from human umbilical cord mesenchymal stem cells, bone marrow mesenchymal stem cells and adipose mesenchymal stem cells in lung cancer, to provide new alternatives for the treatment of lung cancer.

## Introduction

Lung cancer is the most common cancer in the world and the leading cause of cancer-related deaths. Because early-stage lung cancer is asymptomatic, most cases are detected at an advanced stage. The prognosis of patients with advanced lung cancer is poor, and the 5-year relative survival rate is about 5.2% (1). In 2018, there were more than 2 million cases of lung cancer worldwide, with about 1.76 million deaths, which has become a major burden on health care around the world (2). Environmental factors are one of the main risk factors for lung cancer, such as smoking, air pollution and radiation exposure (3). At present, the development of lung cancer treatments mainly includes radiotherapy, chemotherapy, surgery and so on (2). Accumulating evidence suggests that mesenchymal stem cell-derived extracellular vesicles (MSC-EVs) play an important role in the development of lung cancer. One study found that EVs derived from human umbilical cord mesenchymal stem cells (hUCMSCs-EVs) could transfer miR-130b-3p into lung cancer cells and promote the occurrence and development of lung cancer through the FOXO3/NFE2L2/TXNRD1 axis (4). Therefore, MSC-EVs may become a new direction for lung cancer treatment.

## Mesenchymal Stem Cells and Extracellular Vesicles

### Sources of Mesenchymal Stem Cells and Their Regulatory Effects

Mesenchymal stem cells (MSCs) are pluripotent cells derived from mesoderm that exist in bone marrow, umbilical cord tissue, placenta, adipose tissue and other tissues, and MSCs have the potential to differentiate into adipocytes, osteoblasts and chondroblasts (5, 6). They have the characteristics of low immunogenicity, multi-directional differentiation and promote tissue regeneration, which make them play a role in anti-inflammation, promoting regeneration and maintaining the stability of the internal environment (7, 8). MSCs, originally discovered in bone marrow (BM), can now be isolated from many organs or tissues, but their origin remains unclear, and growing evidence suggests that MSCs originate from perivascular cells (5). Isolated pericytes express the same set of cell surface markers as MSCs, and perivascular cells with typical pericyte markers *in vivo* also express a novel adipose-derived stem cell surface-specific marker (9). At present, it was found that MSCs mainly express CD73, CD90 and CD105, and negatively express CD14, CD34, CD45 and HLA-DR5, but the source of MSCs cannot be distinguished based on these (6). A study has shown that the anti-inflammatory and immunomodulatory effects of MSCs are mainly mediated by non-contact ways such as the release of extracellular vesicles (7). Some evidence shows that MSCs, through their paracrine effects and their ability to modify the microenvironment, alter the activity of other cells and affect tumor cells and immune cells (10). MSCs have been found to increase the secretion of matrix metalloproteinase 9 (MMP9) by activating ABL kinase in lung cancer cells, thereby promote the metastasis of lung cancer cells (11). When adipose-derived MSCs were co-cultured with A549 cells, the growth rate and metastasis rate of A549 cells were increased (12). Current clinical trials of MSCs involve hundreds of diseases. Due to the strong heterogeneity of its cell products, the clinical therapeutic effect varies with different product batches. Varieties of mesenchymal stem cells are currently used in clinical trials. They are divided into two categories: adult mesenchymal stem cells, including bone marrow, adipose tissue, peripheral blood, and dental pulp, and neonatal tissue-derived mesenchymal stem cells, derived from placenta, amniotic membrane, and umbilical cord. Bone marrow mesenchymal stem cells (BMSCs) are the most widely used stem cells in clinical trials, but which derived from birth-related tissues may possess remarkable biological properties, such as high proliferative capacity, longevity, and differentiation potential (13). Therefore, the functional optimization and product quality control of mesenchymal stem cells are the current research focus in cell therapy (14).

### Biogenesis and Regulation of Extracellular Vesicles

Extracellular vesicles (EVs) are accessible to most cells and are widely present in human body fluids (15). There are three different types of EVs: endosomes invaginate to form multivesicular bodies, which fuse with the cell membrane to form EVs with a size of 30-100 nm; the cell membrane buds to form microvesicles with a size of 50-1000 nm; the release of membrane substances during apoptosis will produce apoptotic bodies, the size of which is vary from 100 nanometers to several micrometers (16). It contains proteins, RNA and other substances, and has a lipid bilayer membrane structure (15) and is an important medium of intercellular communication which can regulate endothelial cell function (17). The communication methods of EVs are diverse, including activation of surface receptors, phagocytosis, and endocytosis or membrane fusion (18). RNA in EVs includes various biotypes that represent selected fractions of the source cell’s RNA content, mainly small noncoding RNAs, but also fragmented and intact mRNAs, rRNAs, and lncRNAs (19). The transfer of microRNAs (MiRNAs) regulated by EVs has been shown to affect the progression of all types of cancer, including cancer cell invasion and proliferation, as well as drug resistance. It is reported that EVs secreted by human umbilical cord mesenchymal stem cells with high expression of miR-148B-3p inhibit the development of breast cancer, while extracellular vesicles derived from tumor-associated fibroblasts with low expression of miR-320a inhibit cell proliferation and migration of hepatocellular carcinoma (3). Tumor cell-derived lncRNAs in EVs confer aggressive and chemoresistant phenotypes to adjacent counterparts in the tumor microenvironment. They also mediate the interaction between tumor and stromal cells, thereby remodeling the local environment to promote tumor growth and progression (20).

### Biogenesis of EVs and Their Heterogeneity Based on Mesenchymal Stem Cells

MSCs are one of the most EV-producing cells currently known. Phenotypically, MSC-EVs also expressed CD73, CD90, and CD105, while negatively expressed CD14, CD34, or CD11b (6). Some experiments suggest that MSC-EVs can improve inflammatory diseases by modulating immune function (21). MSC-EVs can inhibit T and B lymphocyte proliferation and induce Treg cell population, and reduce TNF-α expression and increase IL-10 expression by affecting the maturation of macrophages (22). A study showed, MSC-EV-miR-146a plays an anti-inflammatory role by reducing the mRNA and protein levels of TNF receptor-associated factor 6 (TRAF6) and IL-1 receptor-associated kinase 1 (IRAK1), inhibiting the phosphorylation of NF-κ B p65 and I κ B α, reducing the expression of pro-inflammatory factors, and increasing the level of IL-10 (23). Gao et al. co-cultured human umbilical cord mesenchymal stem cell exosomes expressing miR-100-5p with eosinophils treated with oxidized low-density lipoprotein and found that the former can inhibit inflammation through the FZD5/Wnt/β-catenin pathway (24). Because cancer cell lines differ in cancer type, stage, mutation, and drug resistance, the effects of MSC-EVs on different cancer cells may be completely opposite (25). It has been found that BMSC-derived EVs (BMSC-EVs) promotes the invasion, proliferation and migration of osteosarcoma cells through lncRNA MALAT1/miR-143/NRSN2/Wnt/β-Catenin axis. BMSC-EVs can transfer MALAT1 into osteosarcoma cells, thus increasing the expression of MALAT1 and NRSN2, reducing the expression of miR-143, and activating Wnt/β-catenin pathway in osteosarcoma cells (26). Besides, Feng et al. demonstrated that BMSC-EVs can transfer miR-375 to cervical cancer cells to reduce MELK expression, and inhibit the occurrence and progression of cervical cancer (27). Experiments have demonstrated that miR-16 in mouse BMSCs can down-regulate the expression of VEGF at the mRNA and protein levels in breast cancer cells and inhibit angiogenesis (25). MSCs-EVs are implicated in many lung pathologies, such as acute lung injury, acute respiratory distress syndrome and lung cancer (2). Wang et al. found that Intratracheal and intravenous administration of MSC-EVs attenuates lipopolysaccharide-induced lung injury by increasing miR-27a-3p levels, reducing NFKB1, and promoting alveolar macrophage M2 polarization (28). MSC-EVs also showed protection in COPD. Through chronic cigarette smoke-induced COPD mice model, Guo et al. found MSC-EVs can improve lung function, and reduce pro-inflammatory cytokine production, the total number of macrophages and neutrophils to reduce inflammation (29). Studies have shown that BMSC-EVs can transfer miR-186 into fibroblasts to stop the cells activation by inhibiting the expression of SOX4 and DKK1, thereby alleviating idiopathic pulmonary fibrosis (30). Gao et al. demonstrated that adipose-derived mesenchymal stem cell-derived EVs (AMSC-EVs) could inhibit PM2.5-induced TGF-βRI by transferring let-7d-5p into recipient cells, thereby alleviating lung fibrosis (31). Liu et al. found that MSC-EVs expressing miR-204 could inhibit the migration and invasion of non-small cell lung cancer through the KLF7/AKT/HIF-1α axis (32). (Additional file 1: **Table S1**).

## Characterization and Isolation of EVs

Currently, there is no globally recognized standardized method for the isolation and purification of EVs, and the method adopted depends on the source of the sample for EV extraction and the volume and application direction of EVs. According to the survey results, the samples for EV isolation and extraction are usually various biological fluids, such as plasma, serum or urine, and conditioned cell culture fluids are also commonly used materials (33). The most common is differential centrifugation. Zhou et al. ultracentrifuged the cells at 300g for 30 minutes, then centrifuge twice at 10,000g for 20 min to obtain EVs. Then, the isolated EVs were washed with 25 ml phosphate buffered saline (PBS), and the supernatant was discarded after spinning again at 100,000 g for 1 h. Finally wash the EV again for immediate use or store at -80°C (30). However, with this method, washing increases the purity, but also leads to a decrease in the number of EVs (34). In addition, sucrose gradient ultracentrifugation is also a very common method. Sucrose concentration gradients can be created using sucrose solutions of different concentrations, including resuspended particles, after centrifugation and PBS dilution to get EV (35). Size exclusion chromatography is increasingly used. First use differential centrifugation to remove cells, debris and apoptotic bodies, then use ultrafiltration to manage the sample volume, and finally use SEC column to separate EVs according to the radius size. The advantages of this method are that the obtained EVs are highly pure and easy to obtain applied to various biological fluids (36). In addition to the above methods, precipitation of EVs by using polyethylene glycol (PEG) is also a good option. Centrifugation followed by mixing with an equal volume of freshly prepared PEG solution can provide EVs of sufficient purity (37). At present, differential centrifugation is still the most commonly used separation method because of its simplicity and economy (38). Finally, the characterization of EVs by different methods is important to evaluate the results of the separation method. The International Society for Extracellular Vesicles (ISEV) recommends quantitative measurement of the source of EVs, as well as to determine the number of EVs as much as possible, and to determine the presence of EV-related components and other non-vesicular, co-separated substances (38) (**Table 1**).

**Table 1:** Advantage or disadvantages of isolation methods of extracellular vesicles.

References	Methods	Advantage	Shortcoming
Konoshenko MY et al. (34) Muraoka S et al. (35) Monguio-Tortajada M et al. (36)	Differential centrifugation Sucrose gradient ultracentrifugation Size exclusion chromatography	Easy operation Low cost Higher purity Higher amounts of EVs protein and RNA Higher purity Easy to obtain	Less quantity Complex operation Time consuming Pollution

## The Role of Extracellular Vesicle Derived From Mesenchymal Stem Cells in Lung Cancer

Studies have shown that MSC-EVs have a bidirectional effect on lung cancer, which can not only promote the migration and invasion of lung cancer cells (39), but also promote the apoptosis of lung cancer cells or inhibit the growth of lung cancer cells (3). EVs from different mesenchymal stem cells have different effects on lung cancer. We mainly introduce the effect of EVs derived from bone marrow mesenchymal stem cells (BMSCs), EVs derived from adipose mesenchymal stem cells (AMSCs), and EVs derived from human umbilical cord mesenchymal stem cells (hUCMSCs) in lung cancer.

### Extracellular Vesicle Derived From Human Umbilical Cord Mesenchymal Stem Cells

A study has shown that hUCMSCs-EVs can reduce the survival rate, migration and invasion ability of lung cancer cells, and promote the apoptosis of lung cancer cells. Xie et al. co-cultured H1299 and H460 cells with hUCMSCs-EVs highly express miR-320a, and found that miR-320a-expressing hUCMSCs-EVs were antitumor both *in vivo* and *in vitro*. They also confirmed that sex-determining region Y-box 4 (Sox4) and miR-320a may have a targeting relationship. Therefore, the hUCMSCs-EVs highly expressing miR-320a may inhibit the growth of lung cancer cells through Sox4 (3). Dong et al. found that miR-410 in hUCMSCs-EVS could be transferred to lung adenocarcinoma cells. They also confirmed that miR-410 directly regulates the expression of the tumor suppressor gene PTEN at the post-transcriptional level, and the expression of PTEN protein decreased in lung adenocarcinoma cells treated with hUCMSCs-EVS, but the expression of PTEN mRNA and protein in hUCMSCs-EVS was not detected. These results suggest that hUCMSCs-EVS can reduce the expression of PTEN protein by transferring miR-410 to lung adenocarcinoma cells, thus regulating the growth of lung adenocarcinoma cells (40). Zhao et al. demonstrated that TGF-β1 in hUCMSCs could affect the promotion of epithelial-mesenchymal transition (EMT), migration and invasion of lung cancer cells by hUCMSCs-EVs through Smad2/3, Akt/GSK-3β, MAPK and NF-κB pathways (39). A study has shown that A549 cells were treated with hUCMSCs-EVs expressing miR-130a-3p, and then detected the content of miR-130a-3p in A549 cells. It was found that the level of miR-130a-3p in A549 cells in the experimental group was significantly increased compared with the control group. At the same time, CCK-8 assay was used to measure cell proliferation, Transwell assay was used to detect cell migration and flow cytometry was used to detect cell apoptosis. The results showed that compared with the control group, the proliferation ability and *in vitro* migration ability of A549 cells in the experimental group were significantly decreased, and the apoptosis rate in both early and late stages was significantly increased (41).

### Extracellular Vesicle Derived From Bone Marrow Mesenchymal Stem Cells

BMSCs play an important role in regulating endogenous processes such as hematopoiesis and tumor survival. Extracellular vesicles derived from bone marrow mesenchymal stem cells (BMSCs-EVs) play a significant role in inhibiting the development of lung cancer and improving patient survival rate (2). Liu et al. detected the expression of let-7i, lysine demethylase 3A (KDM3A), bicorticoid kinase 1 (DCLK1) and ion transport regulator 3 (FXYD3) containing FXYD domain in lung cancer tissues, then determined the regulatory relationship among them, and observed the effects of them on lung cancer cells. At the same time, xenogeneic tumors were transplanted into nude mice to evaluate tumor growth in nude mice. The results showed that LET-7i derived from BMSC-EV suppressed the inhibitory effect of DCLK1 on FXYD3 by down-regulating the expression of KDM3A, thus inhibiting the proliferation, migration and invasion of lung cancer cells (2). Wu et al. have shown that BMSCs-EVs rearrangement miR-193a inhibits colony formation, invasion and migration of cisplatin-resistant non-small cell lung cancer cells by decreasing LRRC1 expression, and promotes apoptosis (42). Through *in vitro* and *in vivo* experiments, Liang et al. found that BMSCs-EVs could downregulate CCNE1 and CCNE2 to inhibit cell proliferation and colony formation in non-small cell lung cancer *via* deliver miR-144 (43). Ren et al. treated A549 and H23 cells with hypoxic or non-hypoxic BMSCs-EVs and found that miR-21-5p could mediate the tumor-promoting effects of hypoxic BMSCs-EVs and the M2-polarizing effect of macrophages. Meanwhile, overexpression of PTEN, PDCD4 and RECK in A549 cells significantly reduced the tumor-promoting effect of miR-21-5p in hypoxic BMSCs-EVs, whereas overexpression of PTEN in monocytes significantly reduced M2 polarization in macrophages. These results confirmed that hypoxic BMSCs-EVs promoted the occurrence and development of non-small cell lung cancer cells and the M2 polarization effect of macrophages through miR-21-5p (7). One study confirmed that after treating A549, H358, H460 and LLC cells with hypoxic BMSC-EVs, hypoxic BMSC-EVs could transfer miR-193a-3p, miR-210-3p and microRNA-5100 into lung cancer cells and activate STAT3-induced EMT, thereby promoting metastasis of lung cancer cells (44). Chen et al. first demonstrated that miR-126-3p could regulate protein tyrosine phosphatase non-receptor type 9 (PTPN9). Then, they co-cultured A549 cells with BMSC-EVs expressing miR-126-3p, and detected the expression of PTPN9 in A549 cells and the effect of miR-126-3p in BMSC-EVs on the occurrence and development of tumor cells, and found that overexpressing miR-126-3p -126-3p BMSC-EVs can inhibit the viability, invasion and migration of non-small cell lung cancer by inhibiting PTPN9 (45). Liu et al. first confirmed that Kruppel-like factor 15 (KLF15) was a target gene of miR-190a-5p using dual-luciferase reporter gene assay, and then detected miR-190a-5p by qRT-PCR and Western blotting the expression regulation of KLF15 and the effect of BMSC-EVs on the migration and invasion of lung cancer cells (A549, LK79, H1975 and HCC827) were detected by Transwell assay. The results showed that BMSC-EVs expressing miR-190a-5p could increase the content of miR-190a-5p in lung cancer cells and inhibit the mRNA and protein expression of KLF15, thereby inhibiting the migration and invasion of lung cancer cells (46).

### Extracellular Vesicle Derived From Adipose Mesenchymal Stem Cells

In recent years, more and more attention has been paid to the study of AMSCs in malignant tumor cells. Some studies have shown that AMSC may be a novel approach for targeted therapy of glioma, and AMSC-derived extracellular vesicles (AMSC-EVs) can increase the efficacy of chemotherapy in hepatocellular carcinoma. Circular RNAs (CircRNAs) have been shown to play critical roles in cell growth and tumor development. Zhang et al. examined the expression of CIRC-100395 in non-small cell lung cancer cells and the interaction among CIRC-100395, miR-141-3p and LATS2, and found that CIRC-100395 in AMSC-EVs could downregulate miR- 141-3p to increase the expression of LATS2, thereby slowing the progression of non-small cell lung cancer. At the same time, they also demonstrated that CIRC-100395 in AMSC-EVs could inhibit the activity of Hippo/YAP signaling pathway in lung cancer cells (8) (**Table 2**).

**Table 2:** Functions of MSC-derived EVs in preclinical models of lung cancer.

Reference	Year	EV type	EV source	Isolation method	Mechanisms
Xie et al.(3)	2021	Exosomes	Human UC-MSCs	ExoQuick ULTRA EV isolation kit (SBI, Palo Alto, CA, USA).	EVs suppress lung cancer cell growth *via* the SOX4/Wnt/β-catenin axis by transferring miR-320a
Dong et al. (40) Zhang et al. (8) Liu et al. (32) Liu et al. (2) Wu et al. (42) Liang et al. (43) Ren et al. (7) Zhao et al. (39) Li et al. (41) Zhang et al. (44) Chen et al. (45) Liu et al. (46)	2018 2021 2021 2021 2020 2020 2019 2018 2021 2019 2021 2020	Exosomes Exosomes Exosomes Exosomes and microvesicles Exosomes Exosomes Exosomes Exosomes Exosomes Exosomes Exosomes Exosomes	Human UC-MSCs Human AMSCs MSCs Human BM-MSCs Human BM-MSCs Human BM-MSCs Human hypoxia pre-challenged BM-MSCs Human UC-MSCs Human UC-MSCs Human hypoxia pre-challenged BM-MSCs Human BM-MSCs Human BM-MSCs	UCF Exosome Isolation Reagent (Geneseed, China) Not available UCF UCF UCF UCF ExoQuick-TC Kit (System Biosciences, CA) UCF UCF UCF UCF	EVs transfer miR-410 to affect the growth of lung cancer cells by inhibiting the expression of PTEN EVs carrying circ_100395 increase LATS2 expression by sponging miR-141-3p to regulate Hippo/YAP signaling pathway, and further inhibit malignant transformation EVs carrying miR-204 inhibit KLF7 expression and AKT/HIF-1α pathway activity, resulting in impaired cell migration, invasion, as well as EMT EVs transferring let-7i inhibit lung cancer progression through the KDM3A/DCLK1/FXYD3 axis EVs shuffle miR-193a to suppress the colony formation, invasion, migration, and proliferation as well as advance apoptosis of lung cancer cells by downregulating LRRC1 EVs transferring miR-144 inhibit cell proliferation, colony formation, and the number of S phase-arrested cells by downregulating CCNE1 and CCNE2 EVs promote lung cancer cell growth and mobility as well as macrophage M2 polarization *via* miR-21-5p delivery EVs induce EMT and enhance the migration and invasion of lung cancer cells, which can be reversed by knock-down of TGF-β1 EVs carrying miR-130a-3p can reduce the proliferation ability and *in vitro* migration ability of lung cancer cells while increasing the rate of apoptosis EVs can promote lung cancer cell invasion by transferring miR-193a-3p, miR-210-3p, and miR-5100 to activate STAT3 signaling-induced EMT EVs overexpressing miR-126-3p can inhibit the viability, invasion and migration of NSCLC by inhibiting PTPN9 EVs carrying miR-190a-5p can inhibit the mRNA and protein expression of KLF15, thereby inhibiting the migration and invasion of lung cancer cells

MSC, mesenchymal stem cells; EVs-extracellular vesicles; UC, umbilical cord; SOX4, sex determining region Y box 4; AMSCs, adipose derived mesenchymal stem cells; LATS2, large tumor suppressor kinase 2; YAP, yes associated protein; KLF7, kruppel like factor 7; EMT, epithelial mesenchymal transformation; BM, bone marrow; UCF, ultracentrifugation; KDM3A, lysine demethylase 3A; DCLK1, doublecortin like kinase 1; FXYD Domain Containing Ion Transport Regulator 3, FXYD domain containing ion transport regulator 3; LRRC1, leucine rich repeat containing 1; CCNE1, Cyclin E1; CCNE2, Cyclin E2; PTEN, phosphatase and tensin homolog deleted on chromosome ten; TGF-β1, transforming growth factor beta 1; STAT3, signal transducer and activator of transcription 3; PTPN9, protein tyrosine phosphatase non-receptor type 9; KLF15, Kruppel-like factor 15.

## Conclusion

At present, we still do not have a specific treatment for lung cancer, to find the relevant molecular targets and target therapy is still the focus of our future research. Many studies have shown that proteins, RNAs, and other substances encapsulated in MSC-EVs can inhibit the growth, migration and drug resistance of lung cancer cells in different ways, which may become a new direction in the treatment of lung cancer. We may be able to regulate the levels of proteins, RNA and other substances in MSC-EVs *in vitro*, especially miRNA, and then transplant MSC-EVs into patients to alleviate the development of lung cancer and prolong the life of patients. However, there are still many problems in the application of exosomes. First, there is no globally unified standardized method for the isolation and purification of EVs, and EVs isolated in different laboratories lead to different experimental results. At the same time, the therapeutic dose and injection time will also have an impact on clinical application (47). Second, how to mass-produce MSC-EVs to meet clinical needs is also a great challenge (48). In addition, the content of different MSC-EVs is heterogeneous (49). In conclusion, it is necessary for us to understand the role and mechanism of MSC-EVs in the occurrence and development of lung cancer, and to determine a globally recognized standardized method for the isolation and purification of EVs as soon as possible. It is believed that MSC-EVs will have broad prospects in the diagnosis and treatment of lung cancer, become new anti-tumor targeted drugs or tumor intervention measures, and bring good news to lung cancer patients.

## Author Contributions

JW: Manuscript writing. YM: Conception and design, final approval of manuscript. YL: Final approval of manuscript. YC: Final approval of manuscript. All authors contributed to the article and approved the submitted version.

## Funding

This work was supported by National Natural Science Foundation of China [No. 81873410, No. 81800043, and No.82070049], and Natural Science Foundation of Hunan Province [No.2020JJ5818].

## Conflict of Interest

The authors declare that the research was conducted in the absence of any commercial or financial relationships that could be construed as a potential conflict of interest.

## Publisher’s Note

All claims expressed in this article are solely those of the authors and do not necessarily represent those of their affiliated organizations, or those of the publisher, the editors and the reviewers. Any product that may be evaluated in this article, or claim that may be made by its manufacturer, is not guaranteed or endorsed by the publisher.
