# Supplementary material for: Extracellular Vesicle Derived From Mesenchymal Stem Cells Have Bidirectional Effects on the Development of Lung Cancer
Source: Front Oncol. 2022 Jul 4;12:914832. doi: 10.3389/fonc.2022.914832 (PMC9289533; doi:10.3389/fonc.2022.914832)
Supplement: Supplementary file 1 [file Table_1.docx]

**Table S1 The effect of MSC-EVs in different diseases**

| References | Year | Mechanisms | Effect |
| --- | --- | --- | --- |
| Liu et al. (23)  Sarhadi VK et al. (26)  Ding et al. (27)  Wang et al. (28)  Zhou et al. (30)  Gao et al. (31) | 2021  2021  2020  2020  2021  2020 | MSC-EVs-miR-146a decreased the expression levels of TRAF6 and IRAK1, inhibited the phosphorylation of NF-κB p65 and IκBα, and increased the level of IL-10  BMSC-EVs decreased the expression of miR-143 and activated the Wnt/β-catenin pathway in osteosarcoma cells by increasing the expression of MALAT1 and NRSN2  BMSC-EVs transfer miR-375 into cervical cancer cells to reduce MELK expression  Intratracheal and intravenous administration of MSC-EVs increases miR-27a-3p levels, reduces NFKB1 and promotes alveolar macrophage M2 polarization  BMSC-EVs inhibit the expression of SOX4 and DKK1 by transferring miR-186  AMSC-EVs inhibit PM2.5-induced TGF-βRI via transfer of let-7d-5p | Anti-inflammatory  Promoting invasion, proliferation and migration of osteosarcoma cells  Inhibiting the occurrence and progression of cervical cancer  Attenuateing lipopolysaccharide-induced lung injury  Alleviating idiopathic pulmonary fibrosis  Alleviating lung fibrosis |
